# Disruptions of default mode network and precuneus connectivity associated with cognitive dysfunctions in tinnitus

**DOI:** 10.1038/s41598-023-32599-0

**Published:** 2023-04-07

**Authors:** Stephanie Rosemann, Josef P. Rauschecker

**Affiliations:** Laboratory of Integrative Neuroscience and Cognition, Department of Neuroscience, Georgetown University Medical Center, 3970 Reservoir Rd NW, Washington, DC 20057 USA

**Keywords:** Cortex, Cognitive neuroscience, Human behaviour

## Abstract

Tinnitus is the perception of a ringing, buzzing or hissing sound “in the ear” without external stimulation. Previous research has demonstrated changes in resting-state functional connectivity in tinnitus, but findings do not overlap and are even contradictory. Furthermore, how altered functional connectivity in tinnitus is related to cognitive abilities is currently unknown. Here we investigated resting-state functional connectivity differences between 20 patients with chronic tinnitus and 20 control participants matched in age, sex and hearing loss. All participants underwent functional magnetic resonance imaging, audiometric and cognitive assessments, and filled in questionnaires targeting anxiety and depression. Significant differences in functional connectivity between tinnitus patients and control participants were not obtained. However, we did find significant associations between cognitive scores and functional coupling of the default mode network and the precuneus with the superior parietal lobule, supramarginal gyrus, and orbitofrontal cortex. Further, tinnitus distress correlated with connectivity between the precuneus and the lateral occipital complex. This is the first study providing evidence for disruptions of default mode network and precuneus coupling that are related to cognitive dysfunctions in tinnitus. The constant attempt to decrease the tinnitus sensation might occupy certain brain resources otherwise available for concurrent cognitive operations.

## Introduction

Tinnitus is mostly perceived as ringing, buzzing or hissing “in the ear” without external stimulation and affects 10 to 25% of the adult population and as many as 45% of the elderly population^1–4^. Chronic tinnitus significantly impacts quality of life and mental health as it comes with increased distress, fatigue, depression and anxiety, and may also result in problems with sleeping and concentration^5,6^.

Previous studies have applied resting-state functional magnetic resonance imaging [fMRI] to explore functional abnormalities in tinnitus patients. During a resting-state measurement, participants do not perform a specific task, but their brains are scanned while they are in a so-called ‘resting-state’. Thereby spontaneous low frequency fluctuations (< 0.1 Hz) in the BOLD signal are assessed to identify resting-state networks^7^. This enables the analysis of functional connectivity—the temporal correlation of the BOLD response time courses of two brain regions that may be anatomically separate but are functionally related. Resting-state functional connectivity is particularly interesting within the framework of tinnitus because the perception of tinnitus can potentially be better determined without a distracting task. Up to 85% of chronic tinnitus patients perceive the tinnitus sensation permanently [though possibly at varying strength] and, therefore, resting-state fMRI measurements seem suitable to capture this perception^8^.

Although there are some studies on changes in resting-state functional connectivity in tinnitus patients, findings are scarce and vary in their outcomes. Briefly, these studies show that tinnitus is related to an atypical functional network comprising auditory and non-auditory brain regions^9^. Further, tinnitus is associated with decreases as well as increases in functional connectivity in frontal, parietal, cerebellar, auditory and limbic regions^9–14^. Similarly, typical resting-state networks, like default mode and dorsal attention networks, as well as their connections with the precuneus, are affected by tinnitus^15–18^. Additionally, tinnitus distress correlated with decreased functional connectivity between middle temporal gyrus and middle frontal gyrus^19^ and with increased functional connectivity between middle frontal gyrus and the precuneus^20^. A recent meta-analysis identified consistently increased resting-state functional connectivity in the insula, middle temporal gyrus, inferior and superior frontal gyri, parahippocampal gyrus and cerebellum, as well as decreased resting-state connectivity in the cuneus und thalamus^21^. Although presenting a relatively heterogeneous picture, these studies suggest an interaction of multiple brain areas and networks that are involved in tinnitus perception, and relate differently to tinnitus distress such as anxiety or depression.

Along alterations in resting-state functional connectivity, previous research provided evidence for cognitive deficits, such as reduced control of attention^22–24^, altered inhibitory control^24–26^, and a decline in general cognitive abilities like short-term memory, concentration and orientation^27^. Hence, it seems that tinnitus affects specifically those cognitive abilities that require executive control of attention and inhibition^28,29^. A failure in top-down cognitive control may result in a diminished ability to switch attention away from the tinnitus signal, and therefore the awareness of the tinnitus signal is maintained or even increased^24^. However, how cognitive abilities are associated with changes in resting-state functional connectivity in tinnitus has not been investigated. A complicating factor in previous research is that many studies did not control for age and hearing loss and thus it is not clear which underlying functional connectivity changes relate to increasing age, hearing impairment or solely to development of chronic tinnitus. Hence, the question of which pathophysiological mechanisms in the human brain are involved in tinnitus, still needs to be answered. Completing the clinical profile of tinnitus patients by investigating cognitive abilities and their relationship to functional brain alterations may play a crucial role in evaluating and advancing tinnitus interventions and treatment options.

The aim of the current study was to investigate resting-state functional connectivity changes associated with the tinnitus signal but also in relation to tinnitus distress and general cognitive abilities. Based on the above results, we hypothesized that tinnitus would disrupt functional resting-state connectivity in auditory cortex, thalamic, limbic, and prefrontal brain regions^9–13^ as well as in typical resting-state networks, like default-mode and dorsal attention networks, as well as their connections with the precuneus^15–18^. Further, we expected that tinnitus patients exhibit deficits in working memory and may also present with general cognitive decline^22–25,27,29,30^. These decreased cognitive abilities may be reflected in decreased resting-state connectivity between the default mode and dorsal attention networks^16,24^. Moreover, we hypothesized that tinnitus distress is related to *decreased* functional resting-state connectivity between right middle temporal gyrus and middle frontal gyrus^19^ and with *increased* functional connectivity between middle frontal gyrus and the precuneus^20^.

## Results

### Behavioral assessments

Mean values for the MoCA were 27.1 (± 2.14) for the tinnitus patients and 26.9 (± 2.37) for the control participants. The mean performance in the two-back task was 89.7 (± 7.6) % in the tinnitus group and 86.6 (± 7.1) % in the control participants. No significant differences between the groups were obtained for any of the cognitive tasks or in anxiety or depression scores (p > 0.1).

The mean values obtained for the tinnitus assessment questionnaires were 20 (± 11) for the THI and 123 (± 74.7) for the TFI. The mean perceived pitch frequency of the tinnitus was 8 kHz (range 3–12,5 kHz, n = 8 perceived the pitch at 8 kHz and n = 7 at 10 kHz) and the mean perceived tinnitus intensity was 5 dB SL. There was no significant correlation in our sample between tinnitus assessment questionnaires and depression or anxiety scores. Similarly, the values in perceived tinnitus pitch and intensity were not related to any of the depression and anxiety scores (p > 0.1) in our sample. Tinnitus duration ranged from half a year to 50 years (mean duration was 15 years) and did not significantly correlate with any of the behavioral scores. Three patients reported a pulsatile tinnitus, the others reported no pulsation. Four patients indicated their tinnitus sounded like wide-band, high-frequency noise; the others indicated they perceived it as a tone. Four tinnitus patients reported unilateral tinnitus, all others reported a bilateral tinnitus sensation.

### Resting-state functional connectivity

For the resting-state functional connectivity analysis, two different analyses were conducted. First, a between-group comparison was computed to determine differences between tinnitus patients and control participants. Second, a linear regression analysis across tinnitus participants investigating the relation between resting-state functional connectivity and cognitive abilities (MoCA score, two-back task performance) as well as tinnitus distress (THI and TFI scores) was conducted.

In the between-group comparison, we did not obtain a significant difference in resting-state functional connectivity between tinnitus patients and control participants.

**Figure 1:**
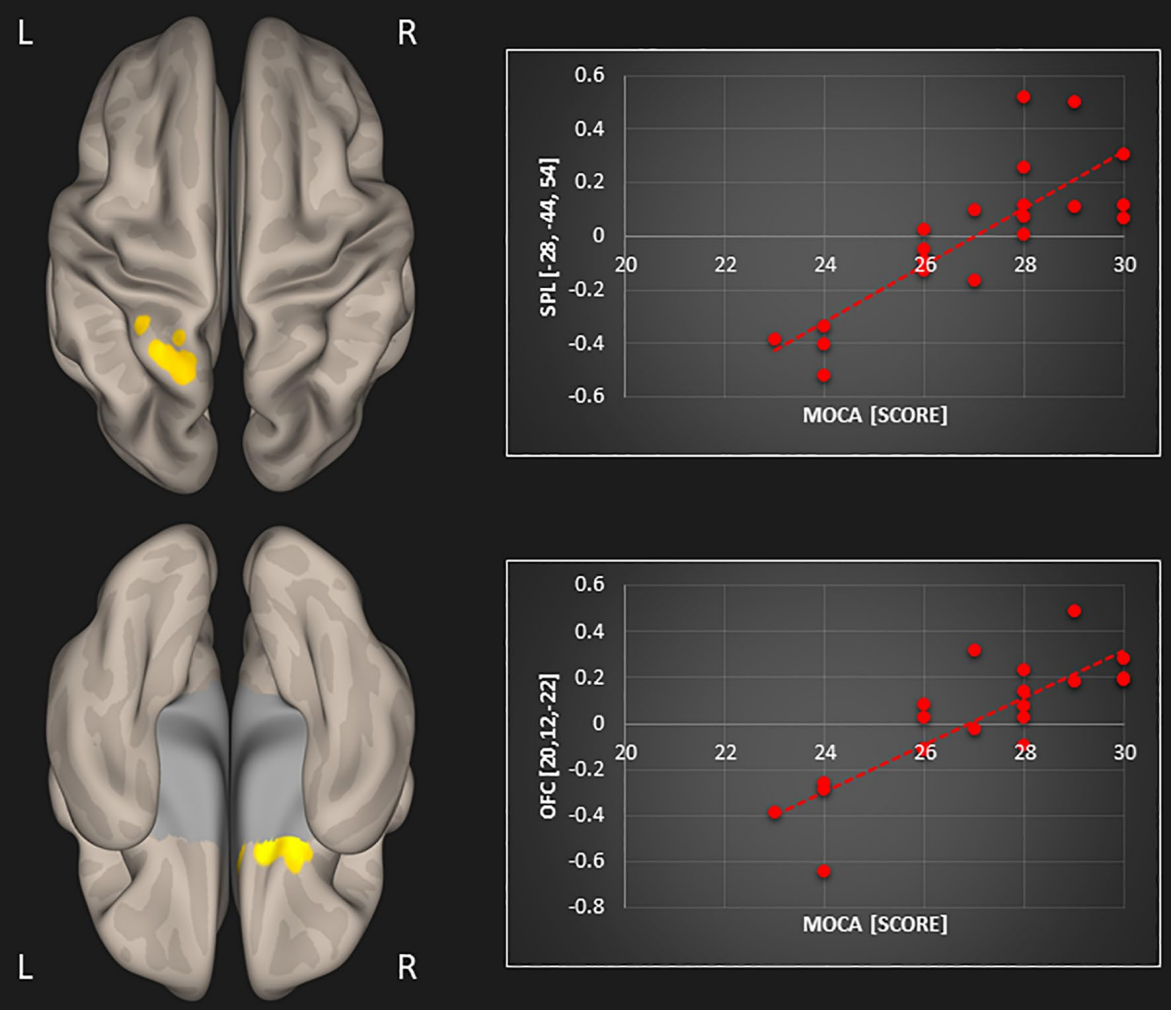
Significant association between MoCA scores and resting-state functional connectivity of the default mode network with the left superior parietal lobule (SPL; top row) and the right orbitofrontal cortex (OFC; bottom row) in tinnitus patients [p < 0.05; FWE cluster-corrected threshold]. Significant brain areas were displayed on an inflated brain by CONN version 20b^31^.

**Figure 2:**
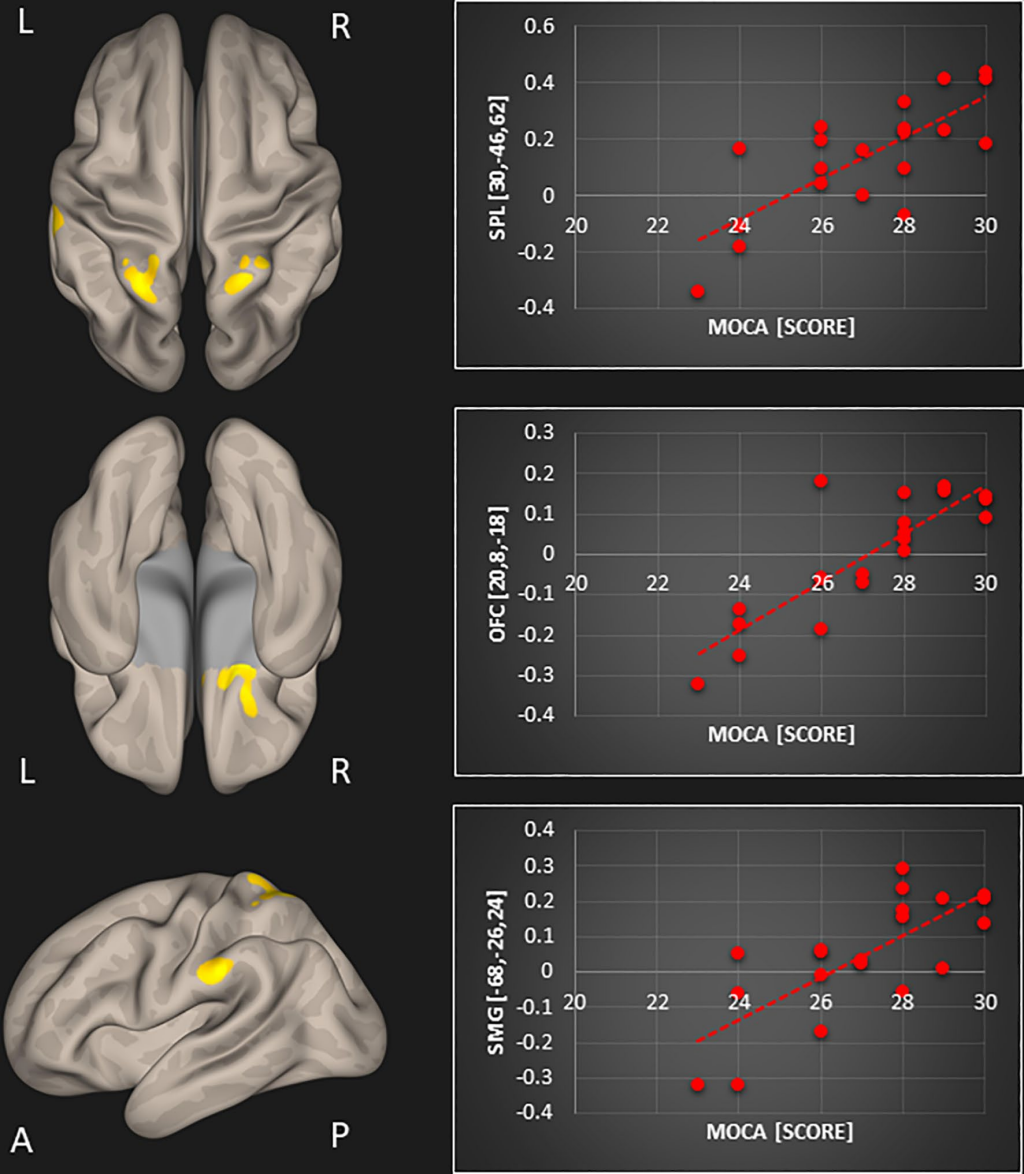
Significant association between MoCA scores and resting-state functional connectivity of the precuneus with the left and right superior parietal lobules (SPL; top row), the right orbitofrontal cortex (OFC; middle row), and the left supramarginal gyrus (SMG; bottom row) in tinnitus patients [p < 0.05; FWE cluster-corrected threshold]. Significant brain areas were displayed on an inflated brain by CONN version 20b^31^.

**Figure 3:**
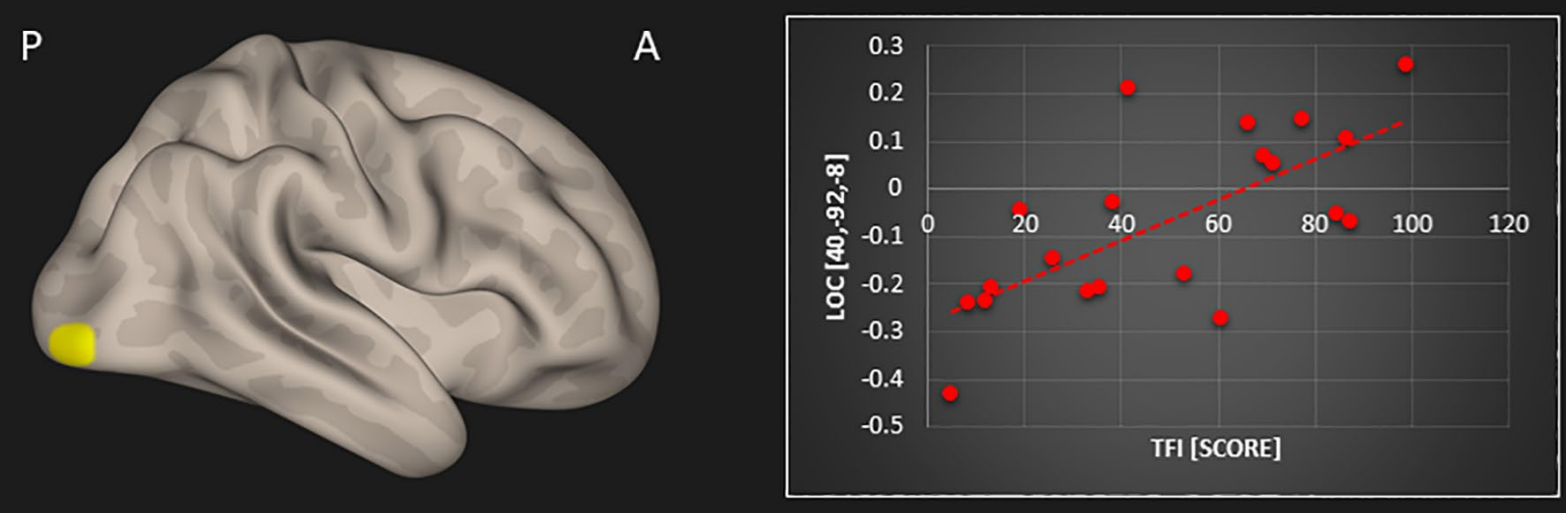
Significant association between TFI scores and resting-state functional connectivity of the precuneus with the right lateral occipital cortex (LOC) in tinnitus patients [p < 0.05; cluster-corrected threshold]. Significant brain areas were displayed on an inflated brain by CONN version 20b^31^.

The subsequent multiple regression analysis in the tinnitus group showed several positive associations between resting-state functional connectivity and MoCA scores as well as TFI scores. An overview including peak coordinates is given in Table 1. First, we obtained a significant positive association between MoCA scores and resting-state functional connectivity of the default mode network and (1) left superior parietal lobule (r = 0.809; p < 0.001) and (2) orbitofrontal cortex (r = 0.825; p < 0.001) in tinnitus patients (Fig. 1). Second, a positive correlation between MoCA scores and resting-state functional connectivity of the precuneus and (1) left (r = 0.754; p < 0.001) and (2) right superior parietal lobule (r = 0.755; p < 0.001), (3) orbitofrontal cortex (r = 0.847; p < 0.001), and (4) supramarginal gyrus (r = 0.749; p < 0.001) was found in tinnitus patients (Fig. 2). Lastly, a significant positive relation between TFI scores and resting-state functional connectivity of the precuneus and the right lateral occipital cortex was apparent in the tinnitus group (r = 0.69; p < 0.001; Fig. 3).

**Table 1:** Resulting clusters of multiple regression within the tinnitus group controlled for age and sex (significant clusters that survive Bonferroni correction are displayed in bold).

Test	Seed region	Peak coordinates (x, y, z)	Z-score	Cluster size	Brain region
MoCA	Default mode network	**− 28, − 44, 54**	**4.04**	**154**	**Superior parietal lobule L**
		20, 12, − 22	4.34	136	Orbitofrontal cortex R
	Precuneus	**20, 8, − 18**	**4.46**	**167**	**Orbitofrontal cortex R**
		**30, − 46, 62**	**4.14**	**147**	**Superior parietal lobule R**
		− 26, − 50, 62	4.22	136	Superior parietal lobule L
		− 68, − 26, 24	4.26	102	Supramarginal gyrus L
TFI	Precuneus	40, − 92, − 8	3.92	144	Lateral occipital cortex R

When correcting for multiple comparisons (Bonferroni correction), only three correlations survived: The correlation between the MoCA and resting-state functional connectivity of (1) the default mode network with the superior parietal lobule and of the precuneus with (2) the orbitofrontal cortex, and (3) with the right superior parietal lobule (this is also indicated in Table 1).

We also computed correlations between cognitive scores and resting-state functional connectivity for the control group. However, no significant correlations were obtained. In order to assess any significant differences between the correlation coefficients of the tinnitus patients and the control participants, we conducted Fisher r-to-z transformations. This analysis demonstrated that correlation coefficients for control participants and tinnitus patients were significantly different from each other for associations of MoCa scores and functional connectivity (all p < 0.05).

## Discussion

The aims of this study were threefold: (1) to assess resting-state functional connectivity changes in tinnitus patients compared to control participants, and to investigate resting-state functional connectivity in relation to (2) the patients’ cognitive abilities and to (3) their tinnitus distress. Based on prior work, our expectations were to find disrupted functional resting-state connectivity in tinnitus patients in the following brain regions: auditory cortex, thalamus, limbic, and prefrontal areas^9–13^, as well as in typical resting-state networks, such as the default mode and dorsal attention networks, along with their connections to the precuneus^15–18^. Further, we assumed that tinnitus patients may exhibit deficits in working memory and general cognitive abilities^22–25,27,29,30^, which may be reflected in decreased resting-state connectivity between the default mode and dorsal attention networks^16,24^. Moreover, we hypothesized that tinnitus distress is correlated with *decreased* functional resting-state connectivity between right middle temporal gyrus and middle frontal gyrus^19^ along with *increased* connectivity between middle frontal gyrus and the precuneus^20^. Indeed, regarding cognitive abilities assessed by the MoCA and tinnitus distress assessed by the TFI, our results showed some positive associations with resting-state functional connectivity in the tinnitus group which were not observed in the control group. We obtained significant positive relations between MoCA scores and resting-state functional connectivity of the default mode network with (1) the left superior parietal lobule and (2) orbitofrontal cortex; and of the precuneus with (3) left and (4) right superior parietal lobule, (5) orbitofrontal cortex and (6) supramarginal gyrus. Further, we were able to demonstrate a significant positive correlation between TFI scores and resting-state functional connectivity of the precuneus and the right lateral occipital cortex in tinnitus patients.

### No differences in resting-state functional connectivity between tinnitus and control participants

Our resting-state functional connectivity analysis did not show any significant differences between tinnitus patients and the control group. This is a surprising finding considering the research that has demonstrated disrupted functional resting-state connectivity in auditory cortex, thalamic, limbic, and prefrontal brain regions as well as in default mode and dorsal attention networks^9–13,15–18^. Possible reasons for the absence of these effects might be a difference in seed regions^10,15^, a higher number of participants in other studies^10,11,15^, groups with unilateral tinnitus (12,16), but also control groups that were not matched for age and hearing loss^9,11,13^. Another possibility is that different analysis methods [seed-to-voxel, ROI-to-ROI, or independent component analysis] may have yielded differing results. In addition, some studies used an “eyes-open” paradigm and others used an "eyes-closed” paradigm for the resting-state acquisition, which might explain some of the heterogeneous findings^32^. It has been previously demonstrated that the choice of paradigm significantly impacts resting-state functional connectivity of visual, auditory and sensorimotor networks^33^. We employed an “eyes-open” approach and our groups were matched in terms of age, sex and hearing loss. However, other patient characteristics, such as extent of hearing loss, tinnitus duration, or severity of tinnitus distress vary across studies^32,33^. The co-occurrence of hyperacusis might play a relevant role as well^34^. A recent study on the Human Connectome Project data showed that functional connectivity of the auditory cortex decreases with age and that tinnitus patients presented even less auditory cortex connectivity than their age-matched controls^35^. Hence, it is essential to carefully match the control groups in future tinnitus research. Another suggestion for future studies might be to include a larger sample size enabling the analysis of specific subtypes of patient groups (for instance, groups with high versus low tinnitus distress). A recent meta-analysis reached a similar conclusion^36^. Furthermore, using similar acquisition paradigms, seed regions and analysis methods across studies to allow comparability and reproducibility would be beneficial.

### General cognitive abilities associated with resting-state functional connectivity of the default mode network and the precuneus

Importantly, we obtained several positive associations between general cognitive abilities assessed by the MoCA and resting-state functional connectivity of the default mode network and the precuneus in tinnitus patients that were not observed in the control participants. The correlations were similar for the default mode network and the precuneus, whose functional connectivity to both orbitofrontal cortex and the superior parietal lobule were associated with general cognitive abilities. Furthermore, resting-state functional connectivity between precuneus and supramarginal gyrus correlated with MoCA values. All of these brain areas have been mentioned in previous tinnitus research. The superior parietal lobule shows less beta activity^37^, reduced connectivity^38^, and altered BOLD responses for visual and auditory attentional tasks in tinnitus patients^39^. Moreover, increased brain activity in the superior parietal lobule has been demonstrated during auditory interference conditions with cognitive conflict in tinnitus patients^26^. The orbitofrontal cortex is a nexus for sensory integration, modulation of autonomic reactions, as well as emotional and reward-related behaviors^40^. It functions as part of a network that includes regions of the medial prefrontal cortex, amygdala, insula, and the dopaminergic midbrain projecting to the ventral and dorsal striatum^40,41^. Thus, this network hub, which is controlled by the orbitofrontal cortex, houses the frontostriatal gating mechanism that has been proposed to reduce the intensity of tinnitus and chronic pain, if it is intact, but enables or exacerbates tinnitus, if it is broken^42,43^. Other studies have demonstrated increased functional connectivity of the orbitofrontal cortex^11,13^ and even found an atypical resting-state network involving the orbitofrontal cortex in tinnitus patients^44^. Moreover, a magnetoencephalographic study revealed differences in global resting-state networks that covered prefrontal, orbitofrontal and parieto-occipital cortex when comparing tinnitus patients to controls^45^. The supramarginal gyrus has been associated with increased responses in an emotional task in chronic tinnitus compared to tinnitus patients with recent onset^46,47^ along with increased regional homogeneity in chronic tinnitus patients^48,49^. Taken together, previous research has suggested that the orbitofrontal cortex, together with the superior parietal lobule and the supramarginal gyrus, might be engaged in compensatory processes attenuating the tinnitus signal. The present study is the first to show a positive relationship between cognitive abilities and changes in resting-state functional connectivity of the default mode network and precuneus to orbitofrontal cortex, superior parietal lobule, and supramarginal gyrus in chronic tinnitus. Decreased coupling was related to lower cognitive scores while tinnitus patients did not show cognitive deficits as a group (mean MoCA score was 27; see “Discussion” below). However, taking a closer look at the correlation between MoCa scores and connectivity, it seems that lower MoCa scores (< 26) are correlated with negative connectivity values (anticorrelation), while higher scores (> 26 indicative of normal cognitive function) are correlated with positive connectivity values. Hence, disruptions of default mode network and precuneus coupling may indeed be related to cognitive dysfunctions in bilateral tinnitus. It is probable, that the constant effort to decrease the tinnitus signal is associated with changes in resting-state functional connectivity. Those resources might no longer be available for other cognitive operations leading to diminished cognitive abilities. Because previous resting-state functional connectivity studies did not assess cognitive abilities, it is unclear whether their findings can be merely attributed to the chronic tinnitus perception or may also be intertwined with cognitive abilities. Further, it was recently shown that there is a bidirectional relationship between tinnitus sensation and cognitive control^22^. Thus, we want to stress the importance of including assessments of cognitive abilities in tinnitus research in the future.

Contrary to our expectations, tinnitus patients did *not* exhibit deficits in working memory or general cognitive abilities as a group, although some tinnitus patients showed decreased general cognitive status indicated by a MoCa score < 26^23–25,27,29,30^. Scores from the attention subtests in the MoCa showed a similar pattern (no impairment, no significant differences from control participants). Unfortunately, the abbreviated form of the trail making test which is included in the MoCa does not include the assessment of processing speed. It is probable, that the complete MoCA score or attention subscores as well as the two-back performance that were used in our study were not targeting the affected cognitive processes in our tinnitus sample. Thus, future research should consider including detailed assessments of attention and attention-switching such as the trail making test, specifically subtests 1 and 5^16^, digit span task^16^, Attention Network Test^23^, a go/no-go task^25^, or a Stroop task^26^. The systematic evaluation of cognitive abilities and clinical characteristics in tinnitus patients along with their association to functional brain changes might also aid in developing or improving treatment options that target their health and wellbeing^50^. Cognitive training or cognitive behavioral therapy might assist tinnitus patients in switching their attention away from the tinnitus sensation and hence reduce tinnitus distress^29,51^. In summary, these results provide a first detailed analysis about the relationship between resting-state functional connectivity of default mode network and precuneus with general cognitive abilities in chronic bilateral tinnitus. The mentioned brain areas should also be considered in neuromodulatory therapies that may aid in restoring functional connectivity of the default mode network and specifically the precuneus.

### Tinnitus distress related to resting-state functional connectivity of the precuneus

Additionally, our results demonstrate a positive relationship between tinnitus distress assessed by the TFI and resting-state functional connectivity between the precuneus and the lateral occipital cortex. Previous research has suggested that *decreased* resting-state functional connectivity of the precuneus may interfere with its underlying functioning and anticorrelation with the dorsal attention network^18^. By contrast, other research has provided evidence of *increased* resting-state functional coupling of the precuneus in chronic tinnitus patients compared to controls^24^ and to patients with high compared to low tinnitus distress^20^. Here we also demonstrate *increased* resting-state functional connectivity between the precuneus and the lateral occipital cortex (a higher-order visual region) with increased tinnitus distress. Similarly, we showed in a previous study that increased cortical thickness in the precuneus is correlated with increased tinnitus distress^52^. Hence, neuroanatomical as well as functional changes in the precuneus might be primarily related to increased distress from tinnitus and thereby an increased effort to decrease it. The precuneus, a medial parietal region with widespread cortical and subcortical connections, plays a central role in the modulation of conscious processes and has been found to be activated during various forms of imagery^53^. Enhanced coupling could be a sign of cross-modal plasticity (similar to that seen in deaf or blind patients) attempting to reduce the gain of the tinnitus sensation (as part of a ‘noise cancellation system’ suggested previously^42^). Visual brain regions might be responding to the internal sensation of the tinnitus signal^15^ by trying to reduce involuntary attention to it^21,54^. Alterations in the visual network are, therefore, mostly thought of as effects of the tinnitus rather than a cause of it^33^. Indeed, various other studies have shown cross-modal effects with involvement of visual brain areas in chronic tinnitus^13,15,19,21,38,45,54^. Some studies even suggest that tinnitus can be triggered or modulated by inputs from other sensory modalities or sensorimotor systems such as the visuo-motor system^55,56^. Hence, visual areas may also serve as potential target areas for interventions, for instance neuromodulatory therapies. Possible intervention strategies may also involve attention-demanding visual tasks such as video games since those may aid in alleviating concentration difficulties and reducing tinnitus distress^39^. However, additional research is needed to clarify the role of visual areas in chronic tinnitus.

## Limitations of the study

There are some limitations of the study. First of all, it is relevant to note that in resting-state fMRI studies, the data are collected during continuous scanning, i.e. there is constant scanner noise. This scanner noise might have interfered with the tinnitus perception, as it could have either masked or exacerbated the tinnitus sensation. In addition, stress and anxiety are known to modulate tinnitus loudness and distress. However, we did not assess these possible effects, which could start well in advance of the actual study. To further assess stress and distress levels of the patients, measuring physiological data such as heart rate variability, blood pressure, or respiratory indicators might provide additional information. Moreover, it is unclear whether the observed alterations reflect underlying changes contributing to the cause of tinnitus or whether they arise as a consequence of tinnitus^33^. An answer to this question is difficult to determine solely on the basis of correlational analyses. Further, tinnitus involves a complex interaction of hearing abilities, age, distress, and cognitive components^22^. Longitudinal studies may aid in disentangling this complexity as well as making inferences about causality of neural alterations in chronic tinnitus.

## Conclusion

This is the first study providing evidence of associations between general cognitive abilities assessed by the MoCa and resting-state functional connectivity of the default mode network and the precuneus in tinnitus patients. Decreased coupling of the default mode network and precuneus (a medial parietal region) was observed with the superior parietal lobule, the supramarginal gyrus (in the inferior parietal lobule), and the orbitofrontal cortex, with decreasing MoCa values. Further, tinnitus distress correlated positively with resting-state functional connectivity between precuneus and lateral occipital complex. The orbitofrontal cortex, together with the superior parietal lobule and the supramarginal gyrus, is thought to be engaged in compensatory processes attenuating the tinnitus signal. We argue that disruptions of its resting-state connectivity are related to cognitive abilities in chronic tinnitus, because the affected areas are involved in decreasing the intensity of the tinnitus sensation. Hence, those resources are occupied and no longer available for concurrent cognitive operations. Further, the increased coupling of the precuneus with increased tinnitus distress might be a sign of compensatory mechanisms attempting to decrease the tinnitus sensation (as part of a ‘noise cancellation system’). Based on our results, we want to stress the importance of including assessments of cognitive abilities in tinnitus research in the future. Furthermore, future studies should aim at replicating previous findings by using similar acquisition paradigms, seed regions and analysis methods across studies, which might help in identifying possible biomarkers for tinnitus. This is of crucial importance in order to advance treatment options for tinnitus or evaluating the efficacy of tinnitus interventions.

## Methods

### Participants

20 tinnitus patients and 20 control participants volunteered in the study. The tinnitus patients and control participants were matched in terms of age and sex. Each group comprised 7 female and 13 male participants. The mean age in tinnitus patients was 58.5 (± 9.82) years and the mean age in the control group was 57.7 (± 10.7) years.

The participants were recruited from previous studies in our lab as well as through social networks and local advertisements. The following groups were excluded: pediatric populations, individuals with HIV, individuals with history of seizures or other neurological disorders, with MRI-incompatible implants, with significant ear asymmetries, those with exposure to loud noise 24 h prior to testing, and pregnant women.

### Ethics declarations

The approval for the study was obtained by the Institutional Review Board at Georgetown University, and the study was conducted in accordance with the Code of Ethics of the World Medical Association (Declaration of Helsinki). All participants gave written and informed consent and were paid for participating in this study.

### Audiometric assessment

**Figure 4:**
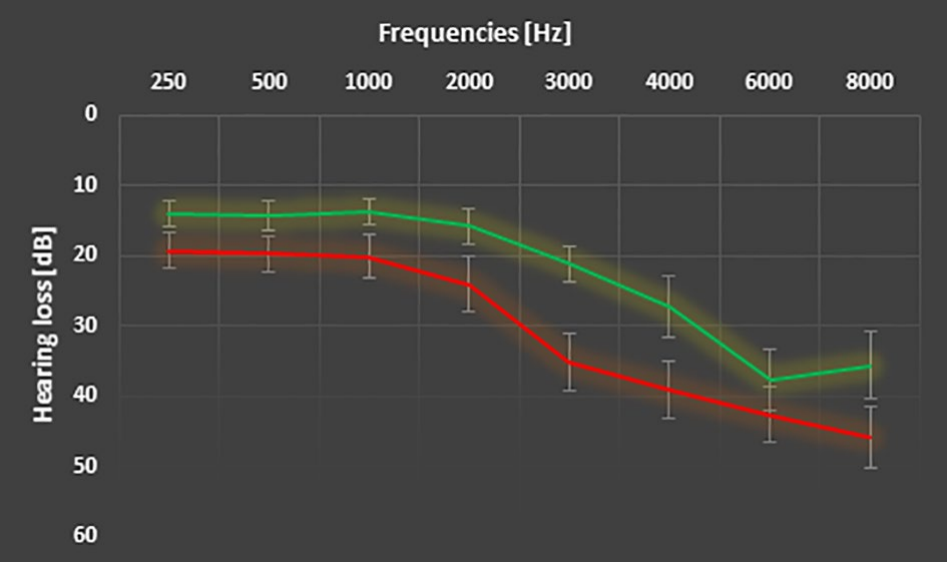
Average pure-tone audiograms for tinnitus patients (red) and control subjects (green) averaged over both ears. Error bars denote standard error of the mean.

Pure-tone thresholds of the frequencies ranging from 250 Hz to 16 kHz were assessed in a soundproof chamber at the Division of Audiology and Hearing Research at Medstar Georgetown University Medical Center. Pure-tone audiograms averaged over both ears for the two groups are depicted in Fig. 4. Hearing thresholds at 3 kHz and 4 kHz as well as the mean hearing loss between 250 Hz and 8 kHz (T(38) = 2.194, p = 0.034) differed significantly between tinnitus patients and control subjects. Hearing thresholds were therefore included in the between-group analysis (see “Data analysis”).

### Behavioral assessment

All participants filled in questionnaires assessing anxiety, depression and emotional distress: The Patient Health Questionnaire 9 [PHQ9]^57^, the Generalized Anxiety Disorder Questionnaire [GAD7]^58^, and the Hospital Anxiety and Depression Scale [HADS]^59^. Every participant conducted the Modified Khalfa Hyperacusis Questionnaire as well^60^. Moreover, tinnitus patients completed the Tinnitus Handicap Inventory [THI]^61^, the Tinnitus Sample Case History Questionnaire [TSCHQ]^62^, and the Tinnitus Functional Index [TFI]^63^. In addition, general cognitive abilities were assessed with the Montreal Cognitive Assessment [MoCA]^64^, and a two-back task was conducted as a measure of working memory (including numbers, duration: 10 min).

### Data acquisition

MRI data were acquired by a 3 T whole-body Siemens Magnetom Prisma MRI machine with a 64-channel head coil. Resting-state data were recorded from all participants while fixating a black dot presented centrally on a grey screen. T_2_*-weighted images were acquired with a gradient echo planar imaging (EPI) sequence (530 volumes, TR = 1430 ms, TE = 35 ms, voxel size = 2.0 × 2.0 × 1.8 mm, flip angle 30 degrees, 68 slices). Structural images were acquired with a 3-D T1-weighted sequence (MP-RAGE, TR = 1900 ms, TE = 2.52 ms, voxel size = 1.0 × 1.0 × 1.0 mm^3^, flip angle 9 degrees, 160 sagittal slices).

### Data analysis

Resting-state functional connectivity analyses were done in SPM12 and CONN version 20b^31^ based on Matlab 2020b. Preprocessing in SPM included spatial realignment estimation, slice-time correction, co-registration, normalization to the Montreal Neurological Institute space using parameters obtained from segmentation of the anatomical T1-weighted image and smoothing (full-width-at-half-maximum = 8 mm). This was followed by preprocessing in CONN including detrending, band-pass filtering (0.008–0.09 Hz), functional outlier detection (Artifact detection tools-based scrubbing^65^) and nuisance regression (motion, mean white matter and cerebrospinal fluid). To ensure data quality, a threshold of 30% of invalid/outlier scans detected with ART was set as exclusion criterion. No subject was excluded (mean 0.009 ± 0.015% outlier scans).

For the group analysis, each subject’s seed-to-voxel connectivity maps of a specific seed to the whole brain (Fisher-transformed correlation coefficients) were entered into a second-level analysis. On the group level, between-subject comparisons (tinnitus patients versus control participants) were performed (corrected for hearing loss). Further, a multiple linear regression analysis with values from the cognitive tasks (MOCA and two-back task) and for tinnitus distress (THI and TFI) within the tinnitus group was conducted. This analysis was controlled for age and sex because of the inhomogeneous tinnitus group (age range 29–71 years; 7 female and 13 male).

Seed regions included the auditory cortex and the precuneus as well as the default mode network, salience network, and dorsal attention network. For the correlation analysis with tinnitus distress, we further used the right middle temporal gyrus as a seed. The seed areas for the auditory cortex (left and right Brodmann areas 41 and 42) and the right middle temporal gyrus were defined using the Automated Anatomical Labeling (AAL) ROI-Library within the Wake Forest University (WFU) PickAtlas^66–68^. Masks of the default mode, salience and dorsal attention network seeds as well as the precuneus were provided by the atlas implemented in CONN (the FSL Harvard–Oxford atlas was used for cortical and subcortical areas).

All nodes of a network were equally weighted, contributing jointly to the seed network's connectivity. Effects were determined to be significant when passing a threshold of p < 0.05 (FWE cluster size inference with p < 0.001 cluster-forming threshold). Bonferroni-correction was applied to correct for multiple comparisons (i.e. five seeds; for tinnitus distress six seeds). Peak coordinates are reported in MNI space.

## Data Availability

The datasets for this study can be found in the OSF project “Tinnitus as a network problem – plasticity in anatomical and functional connectivity”: https://osf.io/2nse8/. Data analysis was preregistered on OSF on July 20, 2021 and can be found at https://osf.io/xhsm5. A preprint version of this manuscript was published on OSF on https://osf.io/r83nw.
